# The origin of the red emission in n-ZnO nanotubes/p-GaN white light emitting diodes

**DOI:** 10.1186/1556-276X-6-130

**Published:** 2011-02-10

**Authors:** N H Alvi, Kamran ul Hasan, Omer Nur, Magnus Willander

**Affiliations:** 1 Department of Science and Technology (ITN) Campus Norrköping, Linköping University, 60174 Norrköping, Sweden

## Abstract

In this article, the electroluminescence (EL) spectra of zinc oxide (ZnO) nanotubes/p-GaN light emitting diodes (LEDs) annealed in different ambients (argon, air, oxygen, and nitrogen) have been investigated. The ZnO nanotubes by aqueous chemical growth (ACG) technique on p-GaN substrates were obtained. The as-grown ZnO nanotubes were annealed in different ambients at 600°C for 30 min. The EL investigations showed that air, oxygen, and nitrogen annealing ambients have strongly affected the deep level emission bands in ZnO. It was concluded from the EL investigation that more than one deep level defect is involved in the red emission appearing between 620 and 750 nm and that the red emission in ZnO can be attributed to oxygen interstitials (O_i_) appearing in the range from 620 nm (1.99 eV) to 690 nm (1.79 eV), and to oxygen vacancies (V_o_) appearing in the range from 690 nm (1.79 eV) to 750 nm (1.65 eV). The annealing ambients, especially the nitrogen ambient, were also found to greatly influence the color-rendering properties and increase the CRI of the as - grown LEDs from 87 to 96.

## Introduction

Zinc oxide (ZnO) is a direct wide band gap (3.37 eV) semiconductor. In recent years, it has attracted the attention of the research community for a variety of practical applications due to its excellent properties combined with the facility of growing it in the nanostructure form.

At present, ZnO is considered to be a very attractive material because it combines semiconducting and piezoelectric properties and in addition it is transparent, biocompatible, and bio-safe. These unique properties of ZnO makes it as a promising candidate for the next generation of visible and ultra-violet (UV) light-emitting diodes (LEDs) and lasing devices. The visible emission results because ZnO possesses deep level emission (DLE) bands and emit all the colors in the visible region with good color-rendering properties [1-8]. It is important to understand the origin of the emissions related to deep level defects in ZnO for the development of optoelectronic devices with high efficiency.

A number of studies on the optical properties of ZnO nanostructures have suggested that, within the DLE, the green (approximately 500 nm) and red (approximately 600 nm) emissions have originated from oxygen vacancies (V_o_) and zinc interstitial (Zn_i_) [9-14]. Other authors have reported that the green emission can be attributed to both oxygen and zinc vacancies [15,16]. The violet-blue and blue emissions were attributed to zinc interstitial (Zn_i_) and Zinc vacancies (V_zn_), respectively, in the DLE [17-19]. The yellow emission in hydrothermally grown nanorods was attributed to the presence of OH groups on the surface [9]. The formation energy and energy levels of different defects within the DLE have been experimentally studied and calculated by other authors [9,20]. However, the origins of different defect emissions are still not fully understood, and the hypotheses that have been proposed to explain the different defect emissions (violet, blue, green, yellow, orange-red, and red) have been controversial [9,10,21,22]. Therefore, still a considerable interest is being shown in investigating the defect emissions in ZnO in general and, ZnO nanostructures in particular, because of their great potential for optical applications.

The ZnO nanotubes are the best candidates for white LEDs among all of the known oxide semiconductors, and they can be easily grown via chemical and other physical vapor-phase approaches as well [6]. The small footprint and the large surface area-to-volume ratio make the ZnO nanotubes a better candidate for heterojunction white LEDs as compared to thin films. The lattice mismatch can be compensated in view of the favorable stress/strain values observed for ZnO nanotubes as compared to thin films. A notable advantage of nanotube-based LEDs is that each nanotube can act as a wave guide, minimizing the side scattering of light, thus enhancing light emission and extraction efficiency [23]. The GaN has close lattice mismatch with ZnO, and the close lattice match is the main factor that can influence the optical and electrical properties of heterojunctions. Only a few studies focusing on n-ZnO nanotubes, on p-GaN, and on white light emitting diodes (LEDs) are available in the literature [24-26].

Many researchers have investigated the DLEs in ZnO. The optical properties of chemically synthesized ZnO nanorods, post-growth annealed in temperatures ranging from 200 to 800°C, have been studied using photoluminescence measurements. In our investigation, the as-grown nanotubes were annealed at 600°C as this temperature was found to be very effective in modifying the DLEs [9,10,21,27,28]. Previously, the authors have investigated the effect of post-growth annealing treatment on the electroluminescence (EL) of n-ZnO nanorods/p-GaN LEDs. The annealing ambients have the same effect on EL of LEDs, but ZnO nanotube-based LEDs were found to have approximately twice the EL intensity as compared to that of ZnO nanorod-based LEDs [29].

ZnO nanostructures grown by low temperature (<100°C) growth techniques such as aqueous chemical growth (ACG) have low crystal quality with lattice and surface defects. The post-growth annealing is an effective tool to enhance and control the crystallinity and optical properties of ZnO nanostructures [21]. In this article, the EL spectra of LEDs fabricated using the as-grown as well as the ZnO nanotubes annealed in argon, air, oxygen, and nitrogen ambients have been investigated. The results showed that oxygen and nitrogen ambients are very effective on modifying the deep level defects, and that the red emission in ZnO was attributed to the superposition of emissions related to oxygen interstitial and oxygen vacancies in ZnO. The post-growth annealing ambient also strongly influences the color-rendering properties of ZnO nanotubes. We have commercially purchased magnesium-doped p-type GaN with film thickness of 4 μm on sphire substrates from TDI Inc. USA. It has hole concentration of approximately 4 × 10^17 ^cm^-3^.

To obtain the ZnO nanotubes, first, the ZnO nanorods were grown on the p-GaN substrates using the low temperature ACG method, and then these nanorods were chemically etched to get nanotubes. There are many chemical growth methods employed for growing ZnO nanorods. The most common method is the one described by Vayssieres et al. [30]. By using this method, the ZnO nanorods were grown on p-GaN substrate. To improve the quality of the grown ZnO nanorods, the said method was combined with the substrate preparation technique developed by Greene et al. [31]. The grown ZnO nanorods on the p-GaN substrates were etched by placing the samples in 5-7.5 molar KCl (Potassium chloride) solution for 5-10 h at 95°C.

The samples were then annealed in argon, air, oxygen, and nitrogen ambients at 600°C for 30 min. Pt/Ni/Au alloy was used to form ohmic contact with the p-GaN substrate. The thicknesses of the Pt, Ni, and the Au layers were 20, 30, and 80 nm, respectively. The samples were then annealed at 350°C for 1 min in flowing argon atmosphere. This alloy gives a minimum specific contact resistance of 5.1 × 10^-4 ^Ω cm^-2 ^[32]. An insulating photo-resist layer was then spun coated on the ZnO NTs to fill the gaps between the nanotubes with a view to isolate electrical contacts on the ZnO NTs to prevent them from reaching the p-type substrate, thereby helping to prevent the carrier cross talk among the nanotubes. To form the top contacts, the tip of the ZnO NTs were exposed using plasma ion-etching technique after the deposition of the insulating photo-resist layer. Non-alloyed Pt/Al metal system was used to form the ohmic contacts to the ZnO NTs. The thicknesses of the Pt and the Al layers were 50 and 60 nm, respectively. This contact gives a minimum specific contact resistance of 1.2 × 10^-5 ^Ω cm^-2 ^[28]. The diameter of the top contact was about 0.58 mm.

## Results and discussions

**Figure 1:**
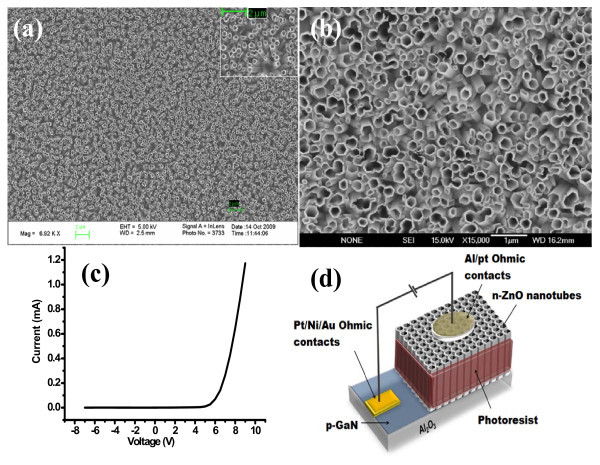
**SEM image of ZnO nanotubes on p-GaN substrate**. **(a) **before annealing, **(b) **after annealing, **(c) **typical I-V characteristics for the fabricated LEDs, and **(d) **The schematic illustration of the fabricated LEDs.

Figure 1a,b shows the images of the top of the ZnO nanotubes before and after annealing, respectively. The figure shows clearly the morphology and size distribution of the as-grown ZnO nanotubes. Hexagonal, well-aligned, vertical ZnO nanotubes were obtained on the p-GaN substrate. The ZnO NTs grown had a uniaxial orientation of 〈0001〉 with an epitaxial orientation with respect to the p-GaN substrate, forming n-ZnO-(NTs)/p-GaN p-n heterojunctions. From the SEM images, the mean inner and outer diameters of the as-grown ZnO nanotubes in this study were found to be approximately 360 and 400 nm, respectively. Figure 1c shows the current-voltage, *I*-*V*, curves of the n-ZnO NTs/p-GaN LEDs developed in this study. All the LEDs have the same *I*-*V *curves. The *I*-*V *curves clearly show a rectifying behavior of the LED as expected with a turn on threshold voltage of about 4 V. This indicates clearly that both metal/GaN and metal/n-ZnO interfaces have formed good ohmic contacts. Figure 1d shows the schematic illustration of the fabricated LEDs.

**Figure 2:**
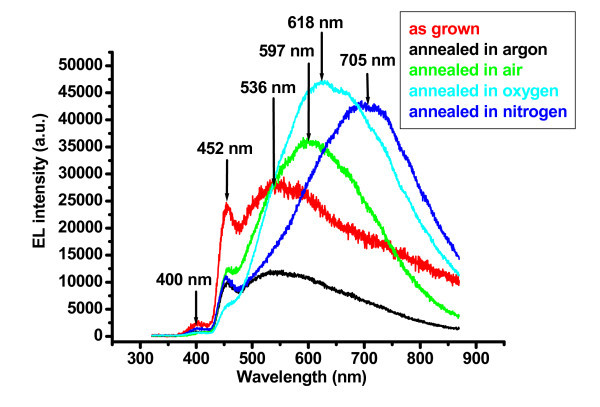
**Electroluminescence spectra of the LEDs at an injection current of 3 mA for the as grown and annealed ZnO NTs in different ambients under forward bias of 25 V and it shows the shift in emission peak after annealing in different ambient**.

Figure 2 shows the EL spectra of the as-grown and annealed LEDs. All the EL measurements were taken under forward bias of 25 V. The EL spectra consist of violet, violet-blue, orange, orange-red, and red peaks. The violet and violet-blue peaks are centered approximately at 400 nm (3.1 eV) and 452 nm (2.74 eV), respectively. The broad green, orange, orange-red, and red peaks are centered approximately at 536 nm (2.31 eV), 597 nm (2.07 eV), 618 nm (2.00 eV), and 705 nm (1.75 eV), respectively. The EL emission in the ultraviolet (UV) region was not detected here since the authors were interested only in the visible emissions; therefore, the lower EL detector limit was set to 400 nm.

The EL intensity of the samples annealed in argon is low compared to the as-grown and all other samples annealed in different ambients. The ZnO nanotubes having low growth temperature (<100°C) possess many intrinsic defects, such as oxygen vacancy (V_o_), zinc vacancy (V_zn_), interstitial zinc (Zn_i_), interstitial oxygen (O_i_), etc., and these defects are responsible for the DLEs. These defects are reduced after annealing at high temperature (600°C). Such activation or passivation of intrinsic defects would greatly enhance the crystal's deep level defect structure leading to the modification of luminescence spectra efficiency of the LEDs [16]. This argument is also confirmed by the EL spectra obtained for ZnO nanotubes annealed in argon (see Figure 2). The EL intensities of the violet (400 nm) and violet-blue (452 nm) of all the annealed samples are decreased as compared with the as-grown samples. In the literature, it was reported that the violet emission from undoped ZnO nanorods is related to Zinc interstitial (Zn_i_) [22]. The violet peak is centered at 3.1 eV (400 nm), and this agrees well with the transition energy from Zn_i _level to the valence band in ZnO (approximately 3.1 eV). The violet-blue peak was centered at 2.74 eV (452 nm) for all the EL measurements in different ambients. It is attributed to recombination between the Zn_i _energy level to the V_Zn _energy level, and approximately is in agreement with the transition energy from Zn_i _energy level to V_Zn _energy level (approximately 2.84 eV). There is a difference of 0.11 eV. This difference maybe is due to the effect of GaN substrate, as GaN also emits blue light. There are no shifts in violet and violet-blue peaks after annealing in different ambients. The violet and violet-blue emissions decreased after annealing the as-grown ZnO nanotubes in different ambients. The violet and violet-blue are the high energy emissions in the visible region, and the annealing affects the deep level defects that are responsible for low energy emissions from the green-to-red region in the visible spectra (see in Figure 2). It increases the transition recombination rate for the deep level defects that are responsible for the green-to-red emissions. Therefore, the EL intensities of the DLEs (the green to red) are increased, while those of the violet and violet-blue emissions are decreased after annealing in different ambients. Only for the case of the argon ambient, all the defects are modified, and owing to this, the El intensities of all the emissions decreased after annealing.

The broad green peak, centered at 536 nm (2.31 eV) in the EL spectra of the as-grown ZnO nanotube-based LEDs and LEDs based on annealed ZnO nanotubes in argon ambient, is attributed to oxygen vacancy (V_o_). It is believed that this phenomenon is due to band transition from zinc interstitial (Zn_i_) to oxygen vacancy (V_o_) defect levels in ZnO [22]. This has been explained by the full potential linear muffin-tin orbital method, which posits that the position of the V_o _level is located approximately at 2.47 eV below the conduction band, and the position of the Zn_i _level is theoretically located at 0.22 eV below the conduction band. Therefore, it is expected that the band transition from Zn_i _to V_o _level is approximately 2.25 eV [22]. This agrees well with the green peak that is centered approximately at 2.31 eV.

The orange-red peaks are centered at 597 nm (2.07 eV) and 618 nm (2.00 eV) for the samples annealed in air and oxygen, respectively. These emissions are attributed to oxygen interstitials O_i_, and believed to be due to band transition from zinc interstitial (Zn_i_) to oxygen interstitial (O_i_) defect levels in ZnO [22]. The position of the O_i _level is located approximately at 2.28 eV below the conduction band, and it is expected that the band transition from Zn_i _to O_i _level is approximately 2.06 eV [22]. This agrees well with the orange-red peaks that are centered approximately at 2.00 and 2.07 eV.

The EL spectra of ZnO nanotubes annealed in oxygen and air ambients are nearly similar. The EL intensity of the sample annealed in oxygen is higher compared to that of the sample annealed in air. Its means that air and oxygen produce the same defects, but the ratio of these defects is more in the case of oxygen. As the orange-red emission is attributed to oxygen interstitials O_i _[22], the annealing in oxygen ambient increases the amount of oxygen-related O_i _defects; therefore, the orange-red emission dominates the EL spectra.

The red emission centered at 705 nm (1.75 eV) can be attributed to oxygen vacancies (V_o_). For the ZnO nanotubes annealed in nitrogen ambient, the following oxygen desorption may occur;

$$\text{ZnO}\;\to \;{\text{V}}_{\text{o}}\;+\;{\text{Zn}}_{\text{Zn}}\;+\;\text{1}/{\text{2O}}_{\text{2}}$$

The zinc vacancies are filled with zinc during the annealing of the ZnO nanotubes in the nitrogen ambient. The majority of defects are oxygen vacancies (V_o_) that are created by the evaporation of oxygen [21]. The red emission centering at 706 nm (1.75 eV) may be attributed to the transition from oxygen vacancy (V_o_) level to top of the valance band in ZnO. Using full-potential linear muffin-tin orbital method, the calculated energy level of the V_o _in ZnO is 1.62 eV below the conduction band [20]. Hence, the energy interval from the V_o _energy level to the top of the valence band is approximately 1.75 eV. It agrees well with that observed for the red emission centered at 1.75 eV.

By comparing the EL spectra of samples annealed in oxygen and nitrogen, it can be concluded that the total red emission ranging from 620 nm (1.99 eV) to 750 nm (1.65 eV) is the combination of emissions related to O_i _and V_o _defects. The EL spectra of the samples annealed in oxygen show that after annealing, the red emission is enhanced in the range from 620 nm (1.99 eV) to 690 nm (1.79 eV) when compared to the as-grown samples, and the EL spectra of the samples annealed in nitrogen ambient show that, after annealing, the red emission is enhanced in the range from 690 nm (1.79 eV) to 750 nm (1.65 eV). The EL intensities of the green, yellow, orange, and the red emission (from 620 to 690 nm) are decreased, but the EL intensity of the red emission (from 690 to 750 nm) has increased significantly as compared with the as-grown ZnO nanotubes. Therefore, it is clear that the red emissions from 620 to 690 nm and from 690 to 750 nm have different origins. The red emission in the range of 620 nm (1.99 eV) to 690 nm (1.79 eV) can be attributed to O_i_, and that in the range of 690 nm (1.79 eV) to 750 nm (1.65 eV) can be attributed to V_o_.

**Figure 3:**
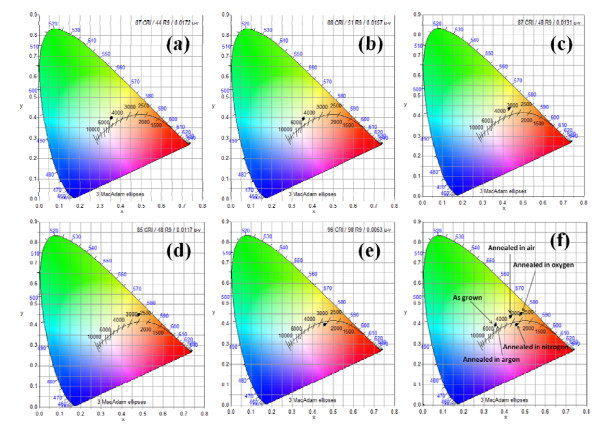
**The CIE 1931 *x*, *y *chromaticity space of ZnO nanotubes**, for **(a) **as grown, **(b) **annealed in argon, **(c) **annealed in air, **(d) **annealed in oxygen, **(e) **annealed in nitrogen, and **(f) **all together.

Figure 3a,b,c,d,e shows the CIE 1931 color space chromaticity diagram in the (*x*, *y*) coordinates system. The chromaticity coordinates are (0.3559, 0.3970), (0.3557, 3934), (0.4300, 0.4348), (0.4800, 0.4486), and (0.4602, 0.3963) with correlated color temperatures (CCTs) of 4802, 4795, 3353, 2713, and 2583 K for the as-grown ZnO nanotubes, annealed in argon, air, oxygen, and nitrogen, in the forward bias, respectively. The chromaticity coordinates are very close to the Planckian locus which is the trace of the chromaticity coordinates of a blackbody. The colors around the Planckian locus can be regarded as white. It is clear that the fabricated LEDs are in fact the white LEDs.

**Figure 4:**
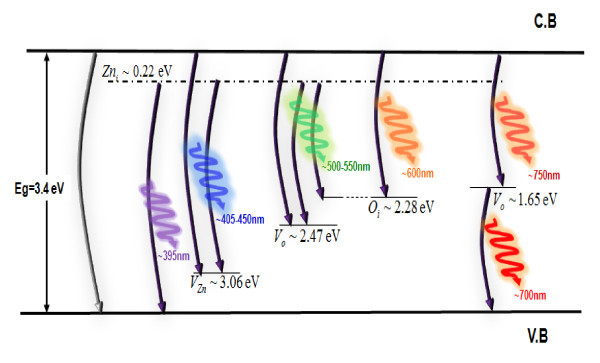
**Schematic band diagram of the DLE emissions in ZnO based on the full potential linear muffin-tin orbital method and the reported data as described in references **[9-20,22]. Also oxygen vacancies situated 1.65 eV below the conduction band are denoted to be contributing to the red emission.

Figure 4 shows the schematic band diagram of the DLE emissions in ZnO, based on the full-potential linear muffin-tin orbital method and the reported data.

In summary, the origin of red emission in chemically obtained ZnO nanotubes has been investigated by EL spectra. The as-grown samples were annealed in different ambient (argon, air, oxygen, and nitrogen). It was observed that the post-growth annealing in nitrogen and oxygen ambients strongly affected the green, yellow, orange, and red emissions of ZnO nanotubes. The EL intensities of the green, the yellow, the orange, and the red emissions were gradually increased after annealing in air, oxygen ambients, and decrease in argon ambient. However, in nitrogen ambient, the EL emission of the red peak in the range of 690--750 nm was increased, and in the range of 620-690 nm, it was decreased as compared with the as-grown samples. It was found that more than one deep level defect are involved in producing the red emission in ZnO.

## Abbreviations

ACG: aqueous chemical growth; DLE: deep level emission; EL: electroluminescence; LEDs: light emitting diodes; UV: ultra-violet; ZnO: zinc oxide.

## Competing interests

The authors declare that they have no competing interests.

## Authors' contributions

All authors contributed equally and read and approved the final manuscript.
